# Effect of vital bleaching on surface roughness and microhardness of nanofilled and nanohybrid composite resins

**DOI:** 10.12688/f1000research.130071.1

**Published:** 2023-02-02

**Authors:** Anindita Chakraborty, Tina Purayil, Kishore Ginjupalli, Kalyana-Chakravarthy Pentapati, Neetha Shenoy

**Affiliations:** 1 Department of Conservative Dentistry and Endodontics, Manipal College of Dental Sciences, Manipal, Manipal Academy of Higher Education, Manipal, Karnataka, 576104, India; 2 Dental Materials, Manipal College of Dental Sciences, Manipal, Manipal Academy of Higher Education, Manipal, Karnataka, 576104, India; 3 Public Health Dentistry, Manipal College of Dental Sciences, Manipal, Manipal Academy of Higher Education, Manipal, Karnataka, 576104, India

**Keywords:** restorative resin, microhardness, surface roughness, in-office bleaching

## Abstract

**Background**: To compare the surface roughness and microhardness of Ceram.x® SphereTEC™ one and Filtek Z350 XT after in-office vital bleaching with Pola office.

**Methods**: A total of 20 samples of 10 mm diameter and 2 mm height of Ceram.x® SphereTEC™ one and Filtek Z350 XT were prepared. The samples were subjected to two bleaching sessions with 35% hydrogen peroxide (Pola office) with a seven-day interval between each session. Surface roughness and microhardness of the prepared samples prior to and after the bleaching regimen were measured using a profilometer and Vickers hardness tester, respectively.

**Results**: A significant reduction (p <0.001) in the surface hardness of Filtek Z350 XT from 26.67 ± 2.10 to 17.83 ± 1.36 Vickers hardness number (VHN) was observed after the bleaching whereas no significant reduction in surface hardness was observed with Ceram.x® SphereTEC™ one. However, in-office bleaching of these materials did not significantly alter their surface roughness.

**Conclusions**: The effect of in-office bleaching on the surface hardness of resin composites seem to vary due to variations in the composition of dental composites such as filler loading. Among the composites tested, in office bleaching of Ceram.x® SphereTEC™ did not result in significant changes in its surface hardness and roughness.

## Introduction

Bleaching removes intrinsic and extrinsic stains from the dental tissues. ^1^^,^^2^ Although the procedure is complex the vast majority work by oxidation, a chemical process that converts organic materials into carbon dioxide and water. The outcome depends on the concentration and the ability of the bleaching agent to reach the chromophore molecules coupled with duration and frequency of contact.

Dental bleaching can be either in-office (under clinical supervision) or at-home bleaching. Bleaching is effective in improving esthetics, but there has been a growing concern in the recent past on the effect of bleaching materials and techniques on existing restorative materials in the oral cavity. With ever-increasing demand for esthetics, there has been an increase in the use of direct esthetic restorative materials, especially dental composites. A dental composite restorative material mainly consists of a polymerizable resin matrix, reinforcing fillers, and a coupling agent that bonds resin with the fillers. The vast majority of dental composites are commercially available for clinical use mainly differ in terms of the resin matrix materials and the fillers used. The clinical performance of these materials significantly vary depending on the type, size, distribution, and concentration of fillers used in the composites.

Ceram.x ^®^ SphereTEC™ one (Dentsply, Konstanz, Germany) is a light-curable nanoceramic, radiopaque restorative material with a granulated filler technology (SphereTEC™). It consists of a blend of pre-polymerized fillers of a size equivalent to 15 μm, non-agglomerated glass of 0.6 μm, and Ytterbium fluoride of 0.6 μm. It has distinctive handling characteristics, natural-looking gloss, and effortless polishing. Its resin matrix consists of a reformed version of the polysiloxane comprising matrix from the original Ceram.x ^®^ mono+/duo+. It is combined with a well-established polyurethane methacrylate, bis-EMA, and TEGDMA to increase its mechanical strength.

Filtek™ Z350 XT (3M, ESPE, St. Paul, USA) universal nanocomposite restorative is a light-activated composite designed for use in anterior and posterior restorations. The nanofillers consist of 20 nm silica and 4–11 nm zirconia, both in combination of non-agglomerated/non-aggregated and aggregated forms. The presence of nanofillers in agglomerated or clustered forms with a broad distribution in the size of the clusters permits higher filler loading as well as superior polishing ability and thus the esthetic characteristics. Both Ceram.x ^®^ SphereTEC™ one and Filtek™ Z350 XT contain fillers in the nanometer range; however, their particle size and distribution is different.

Many studies have reported the action of bleaching agents on restorative materials. ^3^^–^^7^ The observed changes after bleaching of composite resin materials are alterations in smoothness, hardness and reduction in bond strength. ^8^^,^^9^ Such observed changes could be due to the differences in the concentration and type of bleaching agent used. In addition, compositional changes in composites in terms of the resin matrix, filler content, size, and distribution may also affect their susceptibility to bleaching. ^10^ Hence, it is essential to investigate the effect of bleaching on the properties of composites, especially those with newer fillers. The use of hybrid composites as universal composites for anterior and posterior restoration is common during the regular dental practice. In this regard, the present study aimed to compare the surface roughness and microhardness of nanofilled and nanohybrid composite restorative materials subjected to in-office bleaching.

## Methods

A customized split mold was used to fabricate twenty disc-shaped specimens of 10 mm diameter and 2 mm height of Ceram.x ^®^ SphereTEC™ one (Dentsply, Sirona) and Filtek Z350 XT (3M ESPE, St. Paul, USA). Table 1 summarizes information regarding the composition and manufacturers’ details of composite resin materials.

**Table 1.** Composite resin materials used in the study.

Type of composite	Composition		Manufacturer
	Inorganic matrix	Fillers	
Ceram.X SphereTec One	Poly-urethane-methacrylate Bis-EMA TEGDMA	• Spherical, prepolymerized SphereTEC™ fillers (d3,50 ≈ 15 μm) • Non-agglomerated barium glass (d3,50 ≈ 0.6 μm) and ytterbium fluoride (d3,50 ≈ 0.6 μm)	Dentsply, UK
Filtek Z 350XT	Bis-GMA UDMA TEGDMA Bis-EMA PEGDMA	• Non-aggregated 20 nm silica, 4–11 nm zirconia • Aggregated zirconia/silica clusters	3M ESPE, United States

After the composite material was packed into the mold, mylar strip was used both on top and bottom surfaces to obtain a smooth surface on the composite. Subsequently, the composite material was cured for 20 seconds on both sides using a visible light curing unit (3M ESPE Elipar, St Paul, USA).

The prepared discs were subjected to in-office bleaching using 35% hydrogen peroxide (Pola office, SDI Limited, Australia) as per the manufacturer’s instructions. The procedure involved applying a bleaching agent two times with one-week interval between the applications. The bleaching protocol involved the application of a bleaching agent three times onto the surfaces of each disc for 15 mins. The discs were rinsed with distilled water for one minute between each application. After the bleaching process, all the discs were stored in distilled water.

The surface hardness of the discs before and after the bleaching was measured using the Vickers hardness testing machine (MMT X7, Matsuzawa Company, Japan). The specimens were mounted on a platform of the device, and a load of 200 g was applied for 30 seconds. The load was removed after dwell time, and the length of the diagonal of the indentation was measured. Three measurements of each sample were carried out and an average length of the indentation was used for the computation of hardness. The surface hardness was calculated by dividing the load by the area of the indention and was reported as Vickers hardness number (VHN).

Surface roughness of the specimens pre- and post-bleaching was measured using a surface profilometer (Surtronics 3+, Taylor Hobson, UK). The samples were placed on a flat stable surface. The stylus of the profilometer was passed over the surface of the specimen perpendicularly to a distance of 0.8 mm. The experiment was carried out in triplicate on each disc, and average surface roughness, as Ra, was recorded in microns.

### Statistical analysis

All the analyses were done using SPSS version 20 (RRID:SCR_019096). A p-value of < 0.05 was considered statistically significant. Normality was tested using the Kolmogorov Smirnov test. Comparison of mean surface roughness and microhardness before and after the bleaching was done using the Paired t-test. ANCOVA was used to evaluate the significant differences in the surface roughness and microhardness between the materials after adjusting the baseline values. Data for this study can be accessed at Mendeley Data. ^11^

## Results

There was no significant difference in mean microhardness before and after bleaching (p = 0.954) in Ceram.x ^®^ SphereTEC™ one. However, Filtek Z350 XT showed a significant reduction in the surface hardness after bleaching (p < 0.001). There were no significant differences in the mean surface roughness before and after bleaching in both the composite resin materials (p = 0.153 and 0.199), respectively ( Table 2).

**Table 2.** Comparison of microhardness and surface roughness between the composite resin materials before and after bleaching.

	Before	After	P-value
	Mean ± SD	Mean ± SD	
Microhardness (VHN)			
Ceram.x ^®^ SphereTEC™ one	37.59 ± 4.28	37.5 ± 4.34	0.954
Filtek Z350 XT	27.67 ± 2.1	17.83 ± 1.36	<0.001 *
Surface roughness (μm)			
Ceram.x ^®^ SphereTEC™ one	2.66 ± 0.26	2.11 ± 0.92	0.153
Filtek Z 350XT	2.62 ± 0.2	2.52 ± 0.24	0.199

* Denotes statistically significant (p < 0.05), paired t test.

ANCOVA evaluated the difference in microhardness and surface roughness between Ceram.x ^®^ SphereTEC™ one and Filtek Z350 XT after bleaching while adjusting for before bleaching values. The adjusted mean (estimated marginal mean) microhardness after bleaching for Ceram.x ^®^ SphereTEC™ one (35.79 ± 1.45) was significantly higher than Filtek Z350 XT (19.538 ± 1.45) (p < 0.001). However, no significant difference in the adjusted mean (estimated marginal mean) surface roughness after bleaching was seen between Ceram.x ^®^ SphereTEC™ one (2.13 ± 0.2) and Filtek Z350 XT (2.5 ± 0.2) (p = 0.21).

## Discussion

The main objective of the present study was to assess the effect of in-office bleaching on two nanohybrid composites with variations in filler size and loading. As the bleaching process generally affects the surface characteristics of dental composites, both surface roughness and microhardness of Ceram.x ^®^ SphereTEC™ one and Filtek Z350 XT were measured prior to and after bleaching using Pola office. The bleaching agent consisted of hydrogen peroxide/sodium perborate/carbamide peroxide that generally oxidizes the chromophores and improves the shade of the discolored tooth. Exposure of these bleaching materials can also potentially affect the existing restorative materials due to their strong oxidizing ability.

Some of the previous investigations have reported an increase in microhardness of composites after bleaching treatment with carbamide peroxide. ^12^ In contrast, other research studies have indicated a reduction in surface hardness. ^13^ Our study did not show any significant changes in the microhardness and surface roughness concerning nanohybrid composite [Ceram.x ^®^ SphereTEC™ one] which was in accordance with previous reports. ^14^^,^^15^ There was a significant reduction in microhardness of Filtek Z350 XT, whereas the surface roughness remained unaffected. These observations were in agreement with previous research. ^16^ An increase in the surface roughness of restorative materials will facilitate the plaque accumulation on the surface thus affecting the esthetics. ^17^ Similarly, a decrease in the surface hardness makes the material more vulnerable to wear during masticatory force application. ^18^

Hydrogen peroxide tends to cause oxidation, thereby facilitating the generation of free radicals. ^19^ The unreacted double bonds in the polymer resin are prone to oxidative cleavage by peroxides. The by-products of this reaction may bring about a reduction in microhardness. Moreover, the free radicals generated by the peroxides are capable of causing hydrolytic degradation of composite resin at the resin-filler interface, thereby paving the way for filler-matrix debonding, leading to microscopic cracks and thus increasing surface roughness. ^20^

Ceram.x ^®^ SphereTEC™ has a high proportion of filler particles with advanced granulated filler technology. The nanohybrid composition with advanced filler technology ensures a higher filler loading and hence superior flexural strength, compressive strength, and low water sorption. Higher filler loading and reduced resin matrix content reduces the chance of resin matrix oxidation by hydrogen peroxide, making them resilient to acidic bleaching agents. On the other hand, resin composite Filtek Z350 is a nanoparticulated composite compounded by BisGMA, UDMA, BisEMA, and minor proportions of TEGDMA. The overall inorganic filler loading in these composites is about 72% by weight, which is less than Ceram.x ^®^ SphereTEC™ composites with an inorganic filler loading of 77–79% by weight. A low filler loading with a large resin matrix volume makes these composites more prone to oxidation or degradation by bleaching agents, hence a significant reduction in microhardness after bleaching. ^21^

Free radicals induced by peroxides may impact the resin–filler interface and cause a filler–matrix debonding. ^22^ The microhardness of the composites is highly influenced by the amount and type of the inorganic fillers. ^23^ Hence, a reduction in the surface microhardness for Filtek Z350 XT may be due to the inorganic filler loss on the surface. Ceram.x ^®^ SphereTEC™ one has pre-polymerized filler particles of non-agglomerated barium glass and ytterbium fluoride and a resin matrix with highly dispersed methacrylic polysiloxane nanoparticles that are chemically similar to glass or ceramics. Such filler composition is more resistant to abrasion and inorganic filler loss at the surface. Hence no significant changes in microhardness and surface roughness were observed.

## Conclusions

The results of the present study indicate that the effect of in-office bleaching on dental composites vary significantly depending on the type of dental composite. Variations in the composition such as filler size and loading may alter their resistance to bleaching. Hence, the effect of the bleaching agent on the existing composite resin restorations must be considered at the time of selection of the bleaching agent and the regimen for clinical use.

## Data Availability

Mendeley Data: Underlying data for ‘Effect of vital bleaching on surface roughness and microhardness of nanofilled and nanohybrid composite resins’, https://www.doi.org/10.17632/5fjyt8z6vc.1. ^11^ Data are available under the terms of the Creative Commons Attribution 4.0 International license (CC-BY 4.0).
