# Supplementary material for: Effect of in-office bleaching agent on the surface roughness and microhardness of nanofilled and nanohybrid composite resins
Source: F1000Res. 2023 May 18;12:129. Originally published 2023 Feb 2. [Version 2] doi: 10.12688/f1000research.130071.2 (PMC10311121; doi:10.12688/f1000research.130071.2)
Supplement: Peer review history

# Peer review: Reviewer response for version 2

**DOI:** 10.5256/f1000research.148333.r174218

**Published:** 2023-06-29

**Authors:** Osiro Olivia Millicent Awino

**Affiliations:** 1 Department of Dental Sciences, Faulty of Health Sciences, University of Nairobi, Nairobi, Nairobi County, Kenya

The authors have satisfactorily addressed the review comments. The article is scientifically sound and contributes useful information on effects of bleaching on surface characteristics of restorative dental resins. 

 A minor grammatical error to be revised:

1. Introduction, paragraph 2, sentence six - is the word 'that' missing after composites, to read: 'The vast majority of dental resin composites *that* are commercially available...'

Is the work clearly and accurately presented and does it cite the current literature?

Partly

If applicable, is the statistical analysis and its interpretation appropriate?

Yes

Are all the source data underlying the results available to ensure full reproducibility?

Yes

Is the study design appropriate and is the work technically sound?

Yes

Are the conclusions drawn adequately supported by the results?

Partly

Are sufficient details of methods and analysis provided to allow replication by others?

Partly

Reviewer Expertise:

Dental Biomaterials Science

I confirm that I have read this submission and believe that I have an appropriate level of expertise to confirm that it is of an acceptable scientific standard.

# Peer review: Reviewer response for version 2

**DOI:** 10.5256/f1000research.148333.r174219

**Published:** 2023-05-25

**Authors:** Rama Krishna Alla

**Affiliations:** 1 Department of Dental Materials, Vishnu Dental College, Bhīmavaram, Andhra Pradesh, India

I have gone through the feedback provided by the authors and I agree with the information given. You may consider this manuscript to accept for publication in your esteemed journal.

Is the work clearly and accurately presented and does it cite the current literature?

Yes

If applicable, is the statistical analysis and its interpretation appropriate?

Yes

Are all the source data underlying the results available to ensure full reproducibility?

Yes

Is the study design appropriate and is the work technically sound?

Yes

Are the conclusions drawn adequately supported by the results?

Yes

Are sufficient details of methods and analysis provided to allow replication by others?

Yes

Reviewer Expertise:

NA

I confirm that I have read this submission and believe that I have an appropriate level of expertise to confirm that it is of an acceptable scientific standard.

# Peer review: Reviewer response for version 1

**DOI:** 10.5256/f1000research.142799.r168376

**Published:** 2023-04-17

**Authors:** Osiro Olivia Millicent Awino

**Affiliations:** 1 Department of Dental Sciences, Faulty of Health Sciences, University of Nairobi, Nairobi, Nairobi County, Kenya

The article is coherent and addresses a relevant topic. The study design and methodology are suitable to answer the research question.

In the introduction, although a clear description of the relevance of the study is provided together with a description of the composite materials, very little information is covered on bleaching materials and techniques and how these can specifically affect the material properties. For example, where this is attempted in the first two paragraphs, the information is scanty and supported by only two citations. Likewise, in the final paragraph, a general statement is made on the effect of the bleaching agents and suspected attributes regarding the material composition but no specific explanation and corresponding examples which would be helpful in justifying the need for this study. A clear distinction should be made between the nanofilled and the nanohybrid composite.

In the methods, the manufacture of Ceram.X SphereTec One in Table 1 is different from that stated in the introduction.

 The description of the specimens is also lacking important detail - how many specimens were prepared for each composite material? This should also be clarified in the abstract. What was the total sample size and was this calculated based on a determined statistical power?

 What material was the split mould made of? Where was the mylar strip sourced from? 

 How much bleaching agent was applied on the discs? Was it the same quantity for all discs at all times? Were the discs dry at the initial bleaching application, before they were stored in distilled water?

 For the hardness test, the length of indentation was measured but the calculation was derived from the area of indentation. This description should be clarified. 

 The description of the surface roughness measurement is also unclear, specifically, '...passed over the surface of the specimen perpendicularly to a distance of 0.8mm...'

In the results, it would be useful to also report the t-values in Table 2 and F or relevant test values for ANCOVA, as well as the 95% CI.

 Table 2 shows paired t-test results within the same material, that is, before and after for the same material (within) not between the two materials. 

 A statement on the significant difference on microhardness between the two materials should also be captured in the abstract.

In the discussion, reference is made to a study on two nanohybrid composites in the first paragraph - this should be clarified as the title talks of a nanofilled and a nanohybrid. 

 The description of the bleaching agent in the discussion is different from that presented in the methodology - can this have any influence on the outcome observed?

 Although clear arguments are presented in the discussion, some paragraphs lack supporting citations e.g only one in paragraph 4.

 Were there any limitations in this study? Are the conclusions generalizable? Can the observations be accurately and undoubtedly attributed to the type of composite as implied in the conclusion? Do the authors believe that this study was able to show the effect of the bleaching agent on the composite due to the filler type and load?

 Throughout the MS, some minor grammatical errors should also be addressed to improve clarity e.g In the title and objective, is the use of 'vital' correct considering that this was an in vitro study?; In the abstract conclusion, '...seems to vary due to variations...'; In the introduction, the second statement in the first paragraph, '..the majority of bleaching agents...' could improve clarity; in the second paragraph, third statement, '...ever-increasing demand...there has been an increase....'

Is the work clearly and accurately presented and does it cite the current literature?

Partly

If applicable, is the statistical analysis and its interpretation appropriate?

Yes

Are all the source data underlying the results available to ensure full reproducibility?

Yes

Is the study design appropriate and is the work technically sound?

Yes

Are the conclusions drawn adequately supported by the results?

Partly

Are sufficient details of methods and analysis provided to allow replication by others?

Partly

Reviewer Expertise:

Dental Biomaterials Science

I confirm that I have read this submission and believe that I have an appropriate level of expertise to confirm that it is of an acceptable scientific standard, however I have significant reservations, as outlined above.

# Response

**Published:** 2023-05-16

**Authors:** Tina Purayil

**Affiliations:** Manipal University, India

Reviewer 2

Query 1

In the introduction, Although a clear description of the relevance of the study is provided together with a description of the composite materials, very little information is covered on bleaching materials and techniques and how these can specifically affect the material properties. For example, where this is attempted in the first two paragraphs, the information is scanty and supported by only two citations. Likewise, in the final paragraph, a general statement is made on the effect of the bleaching agents and suspected attributes regarding the material composition but no specific explanation and corresponding examples which would be helpful in justifying the need for this study. A clear distinction should be made between the nanofilled and the nanohybrid composite.

 Response 1

 The introduction has been revised.

 Although a clear description of the relevance of the study is provided together with a description of the composite materials, very little information is covered on bleaching materials and techniques and how these can specifically affect the material properties-

 The most commonly used bleaching agents are hydrogen peroxide and carbamide peroxide. These bleaching agents can be applied at-home and in-office and are considered to be effective and relatively safe when supervised by a dentist.

  Many studies have reported the action of bleaching agents on restorative materials. The observed changes after bleaching of composite resin materials are alterations in smoothness, hardness and reduction in bond strength.

 A clear distinction should be made between the nanofilled and the nanohybrid composite.

 Filtek™ Z350 XT (3M, ESPE, St. Paul, USA) is a universal nanocomposite consisting of nanometer-sized filler particles in the composite matrix.

 Ceram.x ^®^ SphereTEC™ one (Dentsply, Konstanz, Germany) is a light-curable nanohybrid composite consisting of nanometer and micrometer-sized fillers with a granulated filler technology (SphereTEC™).

 Query 2

In the methods, the manufacture of Ceram.X SphereTec One in Table 1 is different from that stated in the introduction.

 Response 2

 Manufacturer details of Ceram X Sphertec One in Table 1 has been changed to Dentsply, Konstanz, Germany

 Query 3

 The description of the specimens is also lacking important detail - how many specimens were prepared for each composite material? This should also be clarified in the abstract.

 Response 3

 20 samples each of (10 mm diameter and 2 mm height) Ceram.x® SphereTEC™ one and Filtek Z350 XT were prepared. It has been revised both in abstract and also in the main text.

 Query 4

 What was the total sample size and was this calculated based on a determined statistical power?

 Response 4

 Total sample size is 40.

 Sample size was estimated based on the microhardness values reported by Sharafeddin and Jamalipour which yielded an effect size of 1.42. With a power of 80% and 95% confidence interval, the sample size was estimated to be nine per group

 Query 5

 What material was the split mould made of? Where was the mylar strip sourced from? 

 Response 5

 Split mould was made of stainless steel.

 Manufacturing detail of mylar strip is (SS White Co, Philadelphia, PA, USA) and its been added to the main text

 Query 6

 How much bleaching agent was applied on the discs? Was it the same quantity for all discs at all times? Were the discs dry at the initial bleaching application, before they were stored in distilled water?

 Response 6

 The prepared discs were subjected to 0.1 ml of  35% hydrogen peroxide (Pola office, SDI Limited, Australia)

 Same quantities were used for all the discs.

 Yes, the discs were dry at the initial bleaching application.

 Query 7

 For the hardness test, the length of indentation was measured but the calculation was derived from the area of indentation. This description should be clarified. 

 Response 7

 Using the length of the diagonal of the indentation, area of the indentation [square-shaped indentation] was measured as follows: area of indentation = ½ x length of the diagonal ^2^

 Query 8

 The description of the surface roughness measurement is also unclear, specifically, '...passed over the surface of the specimen perpendicularly to a distance of 0.8mm...'

 Response 8

 The word perpendicular was used in the context that the stylus was moving over the surface We have removed the word “perpendicularly” from the text. Surface roughness of the specimens pre- and post-bleaching was measured using a surface profilometer (Surtronics 3+, Taylor Hobson, UK). The samples were placed on a flat stable surface. The stylus of the profilometer was passed over the surface of the specimen to a distance of 0.8 mm. The experiment was carried out in triplicate on each disc,and average surface roughness, as Ra, was recorded in microns

 Query 9

In the results, it would be useful to also report the t-values in Table 2 and F or relevant test values for ANCOVA, as well as the 95% CI.

 Response 9

 t-values ,F values and 95% CI  have been added to the table 2 and text.

 Query 10

 Table 2 shows paired t-test results within the same material, that is, before and after for the same material (within) not between the two materials. 

 Response 10

 It has been included in the Table 2

 Query 11

 A statement on the significant difference on microhardness between the two materials should also be captured in the abstract.

 Response 11

 This statement is added to the abstract: The adjusted mean (estimated marginal mean) microhardness after bleaching for Ceram.x ^®^ SphereTEC™ one (35.79 ± 1.45) was significantly higher than Filtek Z350 XT (19.54 ± 1.45) (p < 0.001).

 Query 12

In the discussion, reference is made to a study on two nanohybrid composites in the first paragraph - this should be clarified as the title talks of a nanofilled and a nanohybrid. 

 Response 12

 It has been revised in the first paragraph of the discussion: The main objective of the present study was to assess the effect of in-office bleaching on nanohybrid and nanofilled composites

 Query 13

 The description of the bleaching agent in the discussion is different from that presented in the methodology - can this have any influence on the outcome observed?

 Response 13

 The active ingredient of majority of bleaching agents is hydrogen peroxide and generally oxidizes the chromophores and improves the shade of the discolored tooth. It has been revised in the second paragraph of the discussion

 Query 14

 Although clear arguments are presented in the discussion, some paragraphs lack supporting citations e.g only one in paragraph 4.

 Response 14

 Supporting study has been included in paragraph 4 : Preethy NA, Jeevanandan G, Govindaraju L, Subramanian EMG. Comparison of Shear Bond Strength of Three Commercially Available Esthetic Restorative Composite Materials: An In Vitro Study. Int J Clin Pediatr Dent. 2020 Nov 1;13(6):635–9.

 Query 15

 Were there any limitations in this study?

 Response 15

 The results of the present study indicate that compositional variations influence the susceptibility of dental composites to bleaching. However, the present study selected only two types of dental composites. Additional studies on large number of composites and their types (microfilled, nanofilled, hybrid composites etc) may provide more insights on the effect of bleaching on composites restorative materials.

 Query 16

 Are the conclusions generalizable? Can the observations be accurately and undoubtedly attributed to the type of composite as implied in the conclusion? Do the authors believe that this study was able to show the effect of the bleaching agent on the composite due to the filler type and load?

 Response 16

 From the results of the present study, we conclude that the variations in formulation of dental composites can influence their susceptibility to bleaching. In that sense, variations in filler size and loading would also alter the ratio between resin matrix to filler. Hence, the findings of the present study can be generalized to the extent that variations in composition changes their susceptibility to bleaching

 Query 17

 Throughout the MS, some minor grammatical errors should also be addressed to improve clarity e.g In the title and objective, is the use of 'vital' correct considering that this was an in vitro study?; In the abstract conclusion, '...seems to vary due to variations...'; In the introduction, the second statement in the first paragraph, '..the majority of bleaching agents...' could improve clarity; in the second paragraph, third statement, '...ever-increasing demand...there has been an increase....'

 Response 17

 In the title and objective, is the use of 'vital' correct considering that this was an in vitro study?:Title has been rephrased to “Effect of in-office bleaching agent on the surface roughness and microhardness of nanofilled and nanohybrid composite resins”

 In the abstract conclusion, '...seems to vary due to variations..: Abstract has been revised to -In office-bleaching with 35% hydrogen peroxide can reduce the microhardness of nanofilled composite. However, the surface roughness was not influenced by the bleaching procedure in both nanohybrid and nanofilled composite resin materials.

 In introduction-the majority of bleaching agents: Second statement in the first paragraph has been revised to :This procedure involves diffusion of bleaching agent which alters the structure of chromophore molecules present in enamel and dentin , thereby promoting tooth whitening ever-increasing demand...there has been an increase -third statement in the second paragraph has been revised to With greater demand for esthetics, there has been an increase in the use of direct esthetic restorative materials, especially dental composites

# Peer review: Reviewer response for version 1

**DOI:** 10.5256/f1000research.142799.r162247

**Published:** 2023-02-20

**Authors:** Rama Krishna Alla

**Affiliations:** 1 Department of Dental Materials, Vishnu Dental College, Bhīmavaram, Andhra Pradesh, India

This manuscript compared the surface roughness and microhardness of Ceram.x® SphereTEC™ one and Filtek Z350 XT after in-office vital bleaching with Pola office. A total of 20 composite discs with 10 x 2 mm were fabricated with Ceram.x® SphereTEC™ one and Filtek Z350 XT. The samples were subjected to two bleaching sessions with 35% hydrogen peroxide (Pola office) with a seven-day interval between each session. Surface roughness and microhardness of the prepared samples prior to and after the bleaching procedures were measured using a profilometer and Vickers hardness tester, respectively. This study reported a significant reduction (p <0.001) in the surface hardness of Filtek Z350 XT after the bleaching and no significant decrease in surface hardness with Ceram.x® SphereTEC™ one. However, in-office bleaching of these materials did not report a significant change in their surface roughness.

**The detailed report is as follows.**

 This manuscript was well-written and adhered to the standards.

- The introduction was adequate and described the objectives of the study clearly. In the second paragraph, a few references should have been cited to substantiate the information presented.
- The methodology was clearly described. The methodology can include information about the curing lamp specifications and the radiation/wavelength employed.
- Results described well based on the statistical analysis performed.
- The discussion is adequate and consistent with the results. The reasons for varying the surface hardness and roughness were discussed. The outcomes of the current study were compared with those of other comparable investigations. The limitations and the future scope of this study could have been emphasized.
- The conclusions were appropriate based on the results.
- The references were appropriate and mentioned the most recent studies.

The data presented in the current study is useful to the scientific community and clinicians. This study reported a significant decrease in the surface hardness of the composites with the bleaching procedures. This decrease in hardness was dependent on the filler loading to resin ratio. Therefore, the data presented in this study is handy to clinicians for selecting an appropriate composite for their patient's needs.

Is the work clearly and accurately presented and does it cite the current literature?

Yes

If applicable, is the statistical analysis and its interpretation appropriate?

Yes

Are all the source data underlying the results available to ensure full reproducibility?

Yes

Is the study design appropriate and is the work technically sound?

Yes

Are the conclusions drawn adequately supported by the results?

Yes

Are sufficient details of methods and analysis provided to allow replication by others?

Yes

Reviewer Expertise:

Dental Materials, Nanoparticles in dentistry, fibre-reinforced composites.

I confirm that I have read this submission and believe that I have an appropriate level of expertise to confirm that it is of an acceptable scientific standard, however I have significant reservations, as outlined above.

# Response

**Published:** 2023-05-16

**Authors:** Tina Purayil

**Affiliations:** Manipal University, India

Query 1

 The introduction was adequate and described the objectives of the study clearly. In the second paragraph, a few references should have been cited to substantiate the information presented.

 Response 1

 References has been added to the second paragraph.

- Abouassi T, Wolkewitz M, Hahn P. Effect of carbamide peroxide and hydrogen peroxide on enamel surface: an in vitro study. Clin Oral Investig. 2011;15(5):673–80.
- De Geus JL, Wambier LM, Kossatz S, Loguercio AD, Reis A. At-home vs In-office Bleaching: A Systematic Review and Meta-analysis. Oper Dent. 2016 Jul 1;41(4):341–56.

Query 2

 The methodology was clearly described. The methodology can include information about the curing lamp specifications and the radiation/wavelength employed.

 Response 2

 The composite material was cured for 20 seconds on both sides using a visible light curing unit (3M ESPE Elipar, St Paul,MN, USA ) having a light intensity of 1200 wM/cm ^2^.

 Query 3

 The limitations and the future scope of this study could have been emphasized.

 Response 3

 The results of the present study indicate that compositional variations influence the susceptibility of dental composites to bleaching. However, the present study selected only two types of dental composites. Additional studies on large number of composites and their types (microfilled, nanofilled, hybrid composites etc) may provide more insights on the effect of bleaching on composites restorative materials.
